# EVALUATING MMP-2 AND TGFß-RI EXPRESSION IN CIRCULATING TUMOR CELLS OF PANCREATIC CANCER PATIENTS AND THEIR CORRELATION WITH CLINICAL EVOLUTION

**DOI:** 10.1590/0102-672020190001e1433

**Published:** 2019-04-29

**Authors:** José Luiz GASPARINI-JUNIOR, Marcello Ferretti FANELLI, Emne Ali ABDALLAH, Ludmilla Thomé Domingos CHINEN

**Affiliations:** 1 A.C. Camargo Cancer Center, International Research Center, São Paulo, SP, Brazil.

**Keywords:** Neoplastic cells, circulating, Pancreatic neoplasms, Epithelial-mesenchymal transition, Células neoplásicas circulantes, Neoplasias pancreáticas, Transição epitelial-mesenquimal

## Abstract

**Background:**

Metastasis is common in the diagnosis of pancreatic cancer, and the presence of epithelial-mesenchymal transition markers in circulating tumor cells may suggest worse prognosis.

**Aim:**

To correlate the number of circulating tumor cells (CTCs) in the peripheral blood of patients with a locally advanced or metastatic pancreatic tumor and the protein expression involved in epithelial-mesenchymal transition (EMT) in CTCs with clinical characteristics, progression-free survival (PFS) and overall survival (OS).

**Method:**

This was a prospective study conducted using peripheral blood samples collected at three different times. CTCs were quantified by the ISET test and analyzed by immunocytochemistry. Proteins involved in EMT (vimentin, TGFß-RI and MMP2) were analyzed in all CTCs.

**Results:**

Twenty-one patients were included. Median CTCs detected were 22, 20 and 8 CTCs/8 ml blood at baseline, first and second follow-up, respectively. No statistically significant correlation was found in correlating the number of CTCs and the evaluated clinical characteristics, PFS, or OS. There was no difference in PFS and OS among the EMT markers in the groups with and without markers.

**Conclusion:**

CTC analysis was not relevant in this sample for comparing clinical findings, PFS and OS in patients with pancreatic cancer. However, marker analysis in CTCs could be useful for the MMP-2 and/or TGFß-RI expression, as observed by the separate PFS curve.

## RESUMO

**Racional:**

A metástase é comum no diagnóstico de câncer de pâncreas; presença de marcadores de transição epitélio-mesenquimal nas células tumorais circulantes (CTCs) podem sugerir pior prognóstico.

**Objetivo:**

Correlacionar o número de CTCs no sangue periférico de pacientes com tumor de pâncreas localmente avançado ou metastático e expressão de proteínas envolvidas na transição epitélio-mesenquimal (TEM) nas CTCs com características clínicas, sobrevida livre de progressão (SLP) e global (SG).

**Método:**

Estudo prospectivo realizado por meio de coletas de sangue periférico em três tempos distintos. As CTCs foram quantificadas pelo sistema ISET e analisadas por imunocitoquímica. Proteínas envolvidas na TEM (vimentina, TGFß-RI e MMP2) foram analisadas em todas as CTCs.

**Resultados:**

Foram incluídos 21 pacientes. A mediana de CTCs detectadas foi de 22, 20 e 8 CTCs/8 ml de sangue no *baseline, p*rimeiro e segundo *seguimentos*, respectivamente. Na correlação entre número de CTCs e as características clínicas levantadas, SLP, SG não houve correlação estatisticamente significante. Nos marcadores de TEM não houve diferença de SLP e SG entre os grupos que apresentaram e não apresentaram marcação.

**Conclusão:**

As CTCs não se mostraram relevantes na comparação dos achados clínicos, SLP e SG em pacientes com câncer de pâncreas. No entretanto, pode ser que para a análise de marcador seja útil, como observado pelas curvas separadas de expressão de MMP-2 e TGFß-RI nas CTCs.

## INTRODUCTION

Pancreatic adenocarcinoma is a highly aggressive disease. It is the eighth leading cause of cancer death in men and the ninth in women worldwide^15^. Despite these alarming numbers, there has been no sudden change in the mortality rate in the US in the last 80 years, showing that the management and treatment of pancreatic cancer still remains a challenge to this day^9^ ^,^ ^16^.

The spread of cancer can occur through circulating tumor cells (CTC)^13^. The prognostic value of CTCs has been demonstrated in several metastatic tumors such as breast and colorectal, showing that the higher the number of CTCs, the worse the progression-free survival (PFS) and overall survival (OS) of the patients^3^ ^,^ ^12^.

Tumor cells can spread by invading neighboring blood vessels or by using the capillaries formed inside the tumor. In both forms, there is induction of epithelial-mesenchymal transition (EMT)^7^. The molecular mechanism of EMT in tumor progression and cytotoxic drug resistance is not yet fully understood. It is believed that several transcription factors (TWIST, SNAIL, SLUG, ZEB1 and ZEB2) and signaling pathways (Wnt, TGF-β, Hedgehog, Notch and NF-KB) are involved in EMT induction. Among these signaling pathways, TGF-β (Transforming Growth Factor-Beta) plays an important role in pancreatic carcinogenesis in the advanced stages of the disease. TGF-β promotes tumor migration, angiogenesis and invasion, in addition to increasing metalloproteinase (MMP) activity. In pancreatic cancer cell lines, the importance of TGF-ß for inducing EMT has already been shown^2^ ^,^ ^5^ ^,^ ^7^ ^,^ ^10^ ^,^ ^11^ ^,^ ^17^.

Degradation of the extracellular matrix is an essential event for tumor infiltration and dissemination. MMPs are a group of 20 proteases. Among them, high MMP-2 expression is highlighted in pancreatic cancer because it contributes to the development and heterogeneity of this tumor^18^ ^,^ ^19^. Few patients find early-stage pancreatic adenocarcinoma and resistance to cytotoxic drugs is a major factor in the disease progression. 

Therefore, this study aimed to correlate the counting and expression of epithelial-mesenchymal transition genes (vimentin, TGFß-RI and MMP2) in CTCs found in peripheral blood of patients with locally advanced or metastatic pancreatic adenocarcinoma and to correlate these with progression-free survival and overall survival.

## METHODS

### Patients

This was a prospective study conducted at the A.C. Camargo Cancer Center, São Paulo, Brazil. It was approved by the Research Ethics Committee of this institution (CEP Registry No. 1367/10). The sample was recruited by consecutive convenience. Patients with a diagnosis of locally advanced or metastatic pancreatic adenocarcinoma, radiologically proven by computed tomography or magnetic resonance imaging, who were either undergoing systemic treatment or presented tumor progression and who did not undergo any surgical procedure four days prior to collection were included in the study. The clinical data collected from the medical record were: gender, age, TNM tumor staging, serum CA level 19-9 and histological grade. RECIST criteria 1.1^6^ were considered to determine progression.

After signing the informed consent form, three 8 ml samples of peripheral blood were taken from the beginning of the systemic treatment and the other two with intervals of 40 and 80 days from the first collection. This period between collections was proposed taking into consideration that the patient would perform two chemotherapy cycles and would not be in the cytotoxic drug period in the subsequent collection for at least one week. The chemotherapy cycle in which the patient was in was not considered in the second or third collection.

### Collections

Blood sample collections (8 ml) were taken in EDTA tubes. Samples were stored at room temperature for up to 4 h under homogenization and processed in the ISET^®^ system (Rarecells Diagnostics, Paris, France) according to the manufacturer’s instructions. 

Cells were considered CTCs if they have the following criteria: nuclear size equal to or greater than 16 μm, nuclear contour irregularity, presence of visible cytoplasm, high nucleus-cytoplasm ratio (>0.8) and negative labeling for CD45^14^. Images obtained from the results of this technique were done using a white light microscope (Axioskop 40 -Carl Zeiss, Germany) coupled to a digital camera (Sony Cyber-shot DSC-S75). 

### Evaluation of the protein expression of epithelial-mesenchymal transition genes

Immunocytochemistry (ICC) tests were performed on baseline CTCs to verify EMT protein expression. To do so, the following mesenchymal markers were used: vimentin (1:100, Dako, Denmark), MMP-2 (1:100, Sigma-Aldrich, United States) and TGF-ß receptor I (TGFß-RI, 1:100, Sigma -Aldrich, United States).

Each ISET membrane presents 10 spots; each spot was cut and placed on a 24-well plate. After a series of hydration and baths with specific buffers and blocking the endogenous peroxidase with hydrogen peroxide, the first antibody chosen was incubated for 1 h. After this period, Dual Long HRP System (EnvisionTM Fix Dako) was added and stained with DAB (Dako^®^). After staining, the second antibody chosen was incubated for another 1 h and rabbit/mouse (Link) (Kit Envision^TM^ system/AP) and AP Enzyme (Enhancer™) were added. Permanet Red (Dako^®^) was used for the second antibody staining. The spots were then placed on the slides for reading once they were dry.

In addition to cytomorphological analysis, double-labeled immunocytochemistry (DAB +/Permanet Red; Dako^®^) was used using a treatment resistance marker and anti-CD45 (a leukocyte surface marker - 1:100, clone 2B11+PD7/26, Dako^®^) to evaluate and distinguish CTCs from contaminating white blood cells (leukocytes). 

Positive and negative controls were performed for each ICC staining. For controls, blood from the healthy individual enriched with lineage cells suggested by the manufacturer for each antibody was used. The healthy individual’s white blood cells were used as a positive control for CD45, while a spot without antibodies was used for the negative reaction. 

### Statistical analysis

The values of the CTCs observed in each evaluation were categorized according to the median for the statistical analysis. It was assessed according to the median because there is no established cut-off point for CTC count for the pancreatic tumor by the method that was used (ISET). The chi-square test was used to associate demographic and clinical characteristics with categorized CTCs (above or below the median) at each time point. The Kaplan-Meier test was used to estimate the median survival time (overall and progression free). The Log-Rank test was used to compare the risk of death/disease progression between groups. The significance level adopted was 5% and the IBM SPSS version 17 software program was used in the analyses. 

## RESULTS

### Baseline characteristics of the study population

We included 21 patients with locally advanced or metastatic pancreatic adenocarcinoma, 10 men (47.6%) and 11 women (52.4%), with a median age of 67 years (41-84 years). Stage IV of the disease was observed in 14 patients (66.7%) and the predominant histological grade was moderately differentiated, found in eight (38.1%). The main metastasis site was the liver (n=9, 42.9%). The most common therapeutic strategy at the time of inclusion in the study was palliative (n=17, 81%). The baseline characteristics of the patients can be seen in Table 1. 

Three CTC collections were performed with a 40-day interval between them. The median CTCs detected by the ISET system at baseline were 22 CTCs/8 ml of blood (0-194.4). The median was 20 CTCs/8 ml blood (0-175) at the first follow-up and at the second follow-up the median was 8 CTCs/8 ml blood (0-84). There was a loss of one sample at baseline, six baseline losses and one follow-up (four deaths and two dropouts), and three losses between the 1^st^ and 2^nd^ follow-up (two deaths and one dropout). 

**TABLE 1:** Clinical characteristics of the recruited patients.

Characteristic		n	%
Gender	Male	10	47.6
	Female	11	52.4
Age	< 67	9	42.9
	= 67	12	57.1
Stage	II	3	14.3
	III	4	19.1
	IV	14	66.7
Tumor size (T)	2	1	4.8
	3	6	28.6
	4	8	38.1
	No data	6	28.6
Lymph node involvement (N)	0	6	28.6
	1	7	33.3
	No data	8	38.1
Metastasis (M)	0	4	19.1
	1	8	38.1
	No data	9	42.8
Histological grade	Not evaluated	7	33.3
	Well differentiated	2	9.4
	Moderately differentiated	8	38.1
	Slightly differentiated	4	19.4
Antineoplastic chemotherapy therapy	Gemencitabine	6	28.6
	FOLFOX	4	19.0
	FOLFIRI	3	14.3
	FOLFIRINOX	7	33.3
	5-FU	1	4.8
Therapeutic strategy	Adjuvant	1	4.8
	Neo-adjuvant	1	4.8
	Palliative	17	81.0
	Data not available	2	9.4

5-FU=Fluorouracil

### Epithelial-mesenchymal transition

**FIGURE 1:**
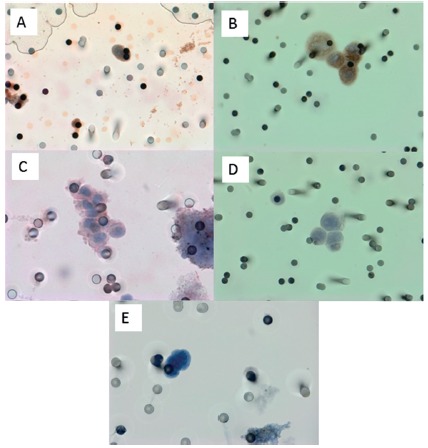
Immunostaining of CTCs and positive controls: A) CTC for pancreatic cancer patient positive for MMP2 (60x); B) positive control, A-549 cell line “spiked” in healthy blood and stained for MMP2 (x60); C) CTC for pancreatic cancer patient positive for TGFβ-RI (60x); D) Positive control, A-549 cell line “spiked” in healthy blood and stained for TGFβ-RI (x60); E) CTC for pancreatic cancer patient stained with hematoxylin (60x). All photomicrographies were taken using a light microscope (Research System Microscope BX61 - Olympus, Tolyo, Japan) coupled to a digital camera (SC100 - Olympus, Tokyo, Japan).

Three markers were used to define the mesenchymal phenotype in the CTCs: vimentin, MMP-2 and TGF-βRI (Figure 1). Six of the 12 patients tested for MMP-2 had positive staining (50%, Figure 1A), while four of the 16 patients tested for TGFß-RI were positive (25%, Figure 1C). Three patients presented positive staining for both markers. None of the 11 tested for vimentin showed any CTC positivity. It should be noted that the antibodies were tested according to the availability of material. Many patients had the ISET membrane spots used in other evaluations from other studies, therefore, they were unavailable for our analyzes. 

**FIGURE 2:**
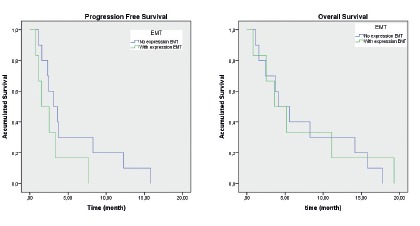
Progression-free survival and overall survival curves for EMT markers between patients who presented (green line) and who did not present (blue line) positive marking in MMP2 and/or TGFβ-RI.

There was no statistically significant difference in PFS among patients who presented TGFß-RI positive staining compared to those who did not present expression of this marker (1.73 months vs. 1.36 months, p=0.83). The same was true for OS (6.95 vs. 6.53 months, p=0.89), with no statistically significant difference (Figure 2). 

Patients with positive staining on MMP-2 CTCs did not present a statistically significant difference in comparison to those without this in PFS (mean: 2, 84 months vs. 4.86 months, p=0.22) and OS (5.82 months vs. 6.39 months, p=0.99). 

Taking into consideration the two markers, patients who presented a positive marker on at least one of the markers (MMP-2 and/or TGFß-RI) in CTCs progressed more than those who did not present positive staining for any of the mesenchymal markers (mean: 2.84 vs. 5.43 months), although there was no statistically significant difference (p=0.14). For OS no difference was observed (5.82 vs. 7.45 months, p=0.65). 

## DISCUSSION

The relevance of CTC analysis in pancreatic adenocarcinoma is controversial. CTCs appear to be good biomarkers because none of the healthy individuals presented CTC in the cohorts of patient´s in which they were analyzed, thus showing their specificity. In addition, the isolation of these cells enables proteomic, genetic and molecular analysis of tumor cells in a non-invasive way^4^ ^,^ ^8^ ^,^ ^14^.

Regarding the expression of molecules involved in EMT, patients who expressed MMP2 and/or TGFß-RI progressed faster (mean: 2.84 vs. 5.43 months, p=0.14) compared to those without mesenchymal phenotype, although the data were not statistically relevant in our study. Some studies corroborate our data in the search for EMT markers. Arumugam et al. (2009)^1^ demonstrated the importance of EMT in cytotoxic drug resistance in pancreatic cancer. The results showed that the five gencitabine-resistant lines (PANC-1, Hs766T, AsPC-1, MIAPaCa-2, MPanc96) showed irregular morphology, high Zeb-1 and vimentin expression, and no E-cadherin expression (p = 0.0006) demonstrating mesenchymal phenotype, unlike gencitabine-sensitive lineages, that showed an epithelial phenotype (HPDE, L3.6pl, BxPC3, CFPAC, SU86.86). In the migration assay, the mesenchymal cells migrated more than the epithelial cells.

Other studies have shown the relevance of CTC as biological markers for EMT in other tumors. Using CTCs collected by the ISET® filtering of patients with head and neck tumors, Fanelli et al. (2017)^8^ demonstrated that the positive labeling for TGFß-RI in CTCs/CTMs (Circulating Tumor Microemboli) was a predictor of worse PFS compared to patients who did not have it in the first follow-up (12 vs. 26 months, respectively, p=0.007). LI et al. (2013)^10^ conducted a study with the objective to evaluate the correlation between CTCs and EMT in hepatic cancer. They used immunofluorescence technique (miniMACS Milteny^®^) in the CTC analysis. The presence of CTC was statistically relevant in patients with tumor thrombosis (p<0.001), stage III and IV of the disease (p=0.005) and tumor size (p<0.001). In those patients who underwent tumor resection or chemoembolization, those with positive CTC had higher tumor recurrence or metastasis (p=0.002) and mortality (p=0.007) than those who did not have CTCs in peripheral blood one year after the first collection. The vimentin and TWIST expression were statistically significant in patients with portal thrombosis per tumor (p=0.0003, p<0.001, respectively). In addition, vimentin expression was also correlated to the largest tumor size (p<0.001). In the immunohistochemical analysis, the vimentin, TWIST and ZEB1 expression were significantly higher in the liver tumor than in other liver diseases (p=0.002, p<0.001, p=0.016, respectively). Tumors expressing E-cadherin (p=0.001) did not express vimentin or TWIST (p=0.020), and E-cadherin expression was low in patients with portal thrombosis per tumor (p=0.007), demonstrating the importance of EMT in spreading tumor cells from the primary tumor. 

Our study was the first to verify the role of TGFß-RI and MMP-2 in CTCs from patients with pancreatic cancer, showing the relevance of MMP-2 expression in these cells. We know the following limitations in our study: number of patients recruited, sample loss among collections, patients at different disease stages, and difficulties encountered in sample analysis (blood coagulation and lack of CTC in all test spots). However, we emphasize the importance of continuing the evaluation of these markers in pancreatic cancer because it is a highly complex disease with so little knowledge about the mechanisms and possibilities of therapeutic intervention. 

## CONCLUSION

The CTC count was not relevant in the comparative analyzes of the number of CTC and PFS, OS or clinical characteristics of the studied population; but, maybe, protein analysis in CTCs can be useful, as noted by the separate PFS curves for MMP-2 and/or TGFß-RI expression.
